# Supplementary material for: Hsp70 Negatively Regulates Autophagy via Governing AMPK Activation, and Dual Hsp70-Autophagy Inhibition Induces Synergetic Cell Death in NSCLC Cells
Source: Int J Mol Sci. 2024 Aug 22;25(16):9090. doi: 10.3390/ijms25169090 (PMC11354248; doi:10.3390/ijms25169090)

Hsp70 negatively regulates autophagy via governing AMPK ac-tivation and dual Hsp70-autophagy inhibition induces syner-getic cell death in NSCLC cells

Bashar Alhasan*, Yana A. Gladova, Dmitry V. Sverchinsky, Nicolai D. Aksenov, Boris A. Margulis, Irina V. Guzhova

Laboratory of Cell Protection Mechanisms, Institute of Cytology, Russian Academy of Sciences, St-Petersburg, 194064, Russia;

*** Corresponding author:** Bashar Alhasan alhasan94@incras.ru

Supplementary materials

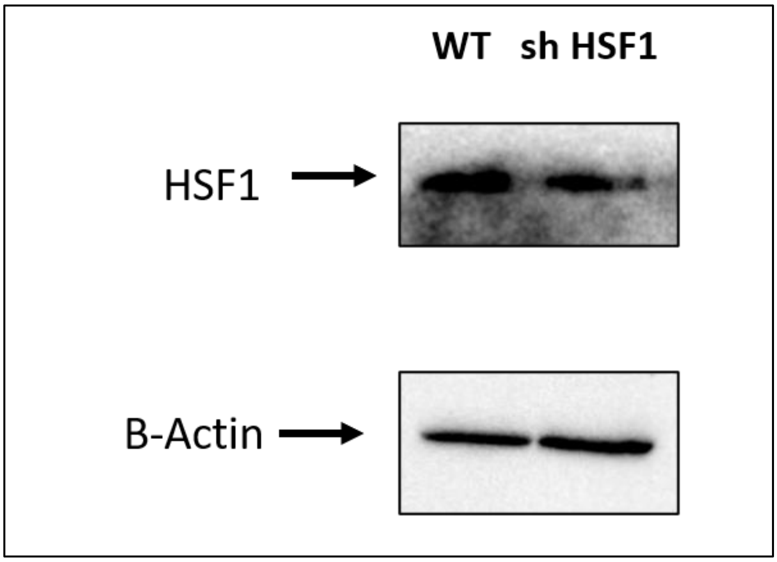

Figure S1. *Confirmation of HSF1 knockdown in A549 tumor cells.*

A549 (sh-scrambled) and A549 (shHSF1) were cultivated until 70-80% confluency, then lysed and subjected to immunoblotting with antibodies against HSP70

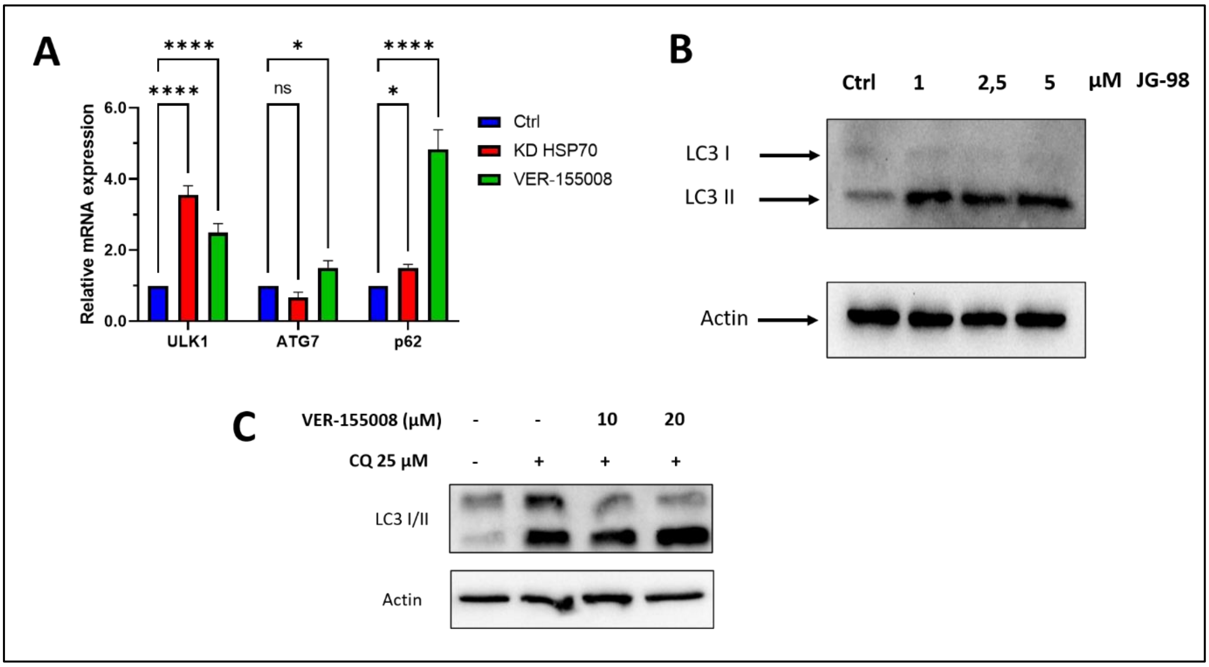

Figure S2. H*sp70 genetic or chemical inhibition upregulates autophagy, both at transcriptional and protein levels in NSCLC tumor cells.*

(A) mRNA samples of A549 control cells, following treatment with 20 µM of VER-155008 for 24 hours, and A549 (shHsp70), were extracted, converted to cDNA and subjected to RT-PCR with primers toward ULK1, ATG7 and p62. Values are the means ± SEM from three independent experiments; * $p < 0.05$, ** $p < 0.01$, *** $p < 0.001$; **** $p < 0.0001$. **(B)** A549 tumor cells were treated with JG-98 at the indicated concentrations for 24 hours, then cells were lysed and subjected to immunoblotting with antibodies against LC3 I/II. **(C)** A549 tumor cells were treated 25 µM of CQ alone or in combination with VER-155008 at the indicated concentration, and then cells were subjected to western blotting to detect LC3 I/II levels.

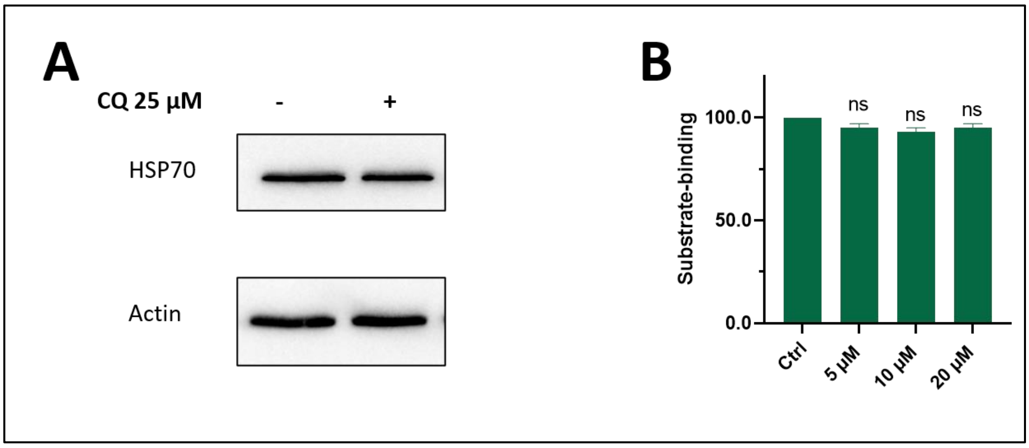

Figure S3. *Autophagy inhibition by Chloroquine doesn't significantly affect Hsp70 expression levels or function in A549 tumor cells*

(A) A549 tumor cells were treated with (25 µM) of Chloroquine for 24 hours and then lysed and subjected to immunoblotting with antibodies against Hsp70. **(B)** A549 tumor cells were treated with CQ at the specified concentration, then they were lysed and subjected to Hsp70 substrate-binding assay. Values are the means ± SEM from three independent experiments; ns: non-significant.

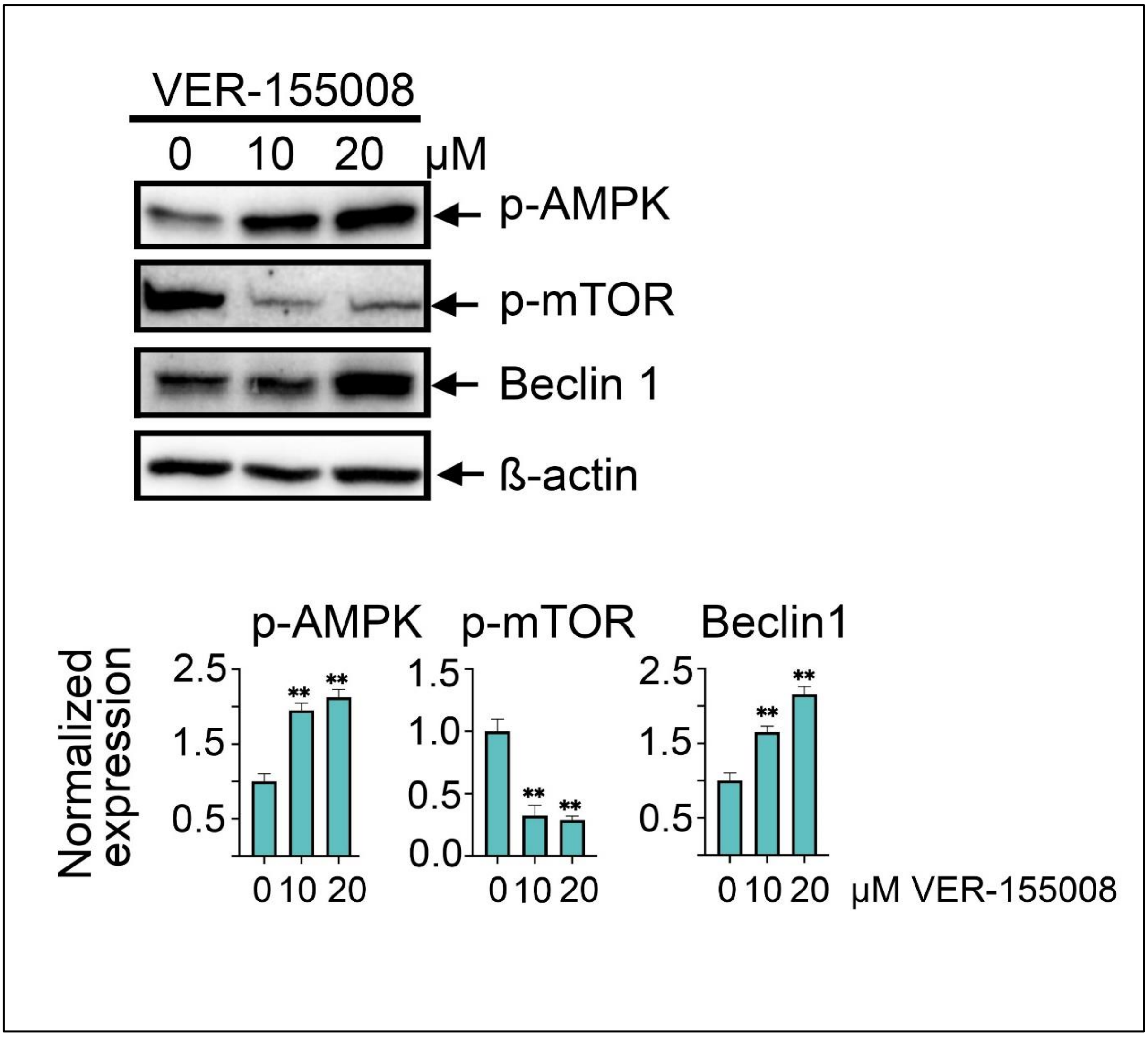

Figure S4. *VER-155008 treatment inhibits mTOR and upregulates AMPK phosphorylation and Beclin1 in H1299 tumor cells.*

H1299 tumor cells were treated with VER-155008 at the indicated concentrations and then were lysed for immunoblotting. Values on the charts represent the relative protein expression, which indicates the ratio between the band intensity of the protein of interest to the band intensity of β-actin. Band intensity was measured using ImageJ program. * $p < 0.05$, ** $p < 0.001$.

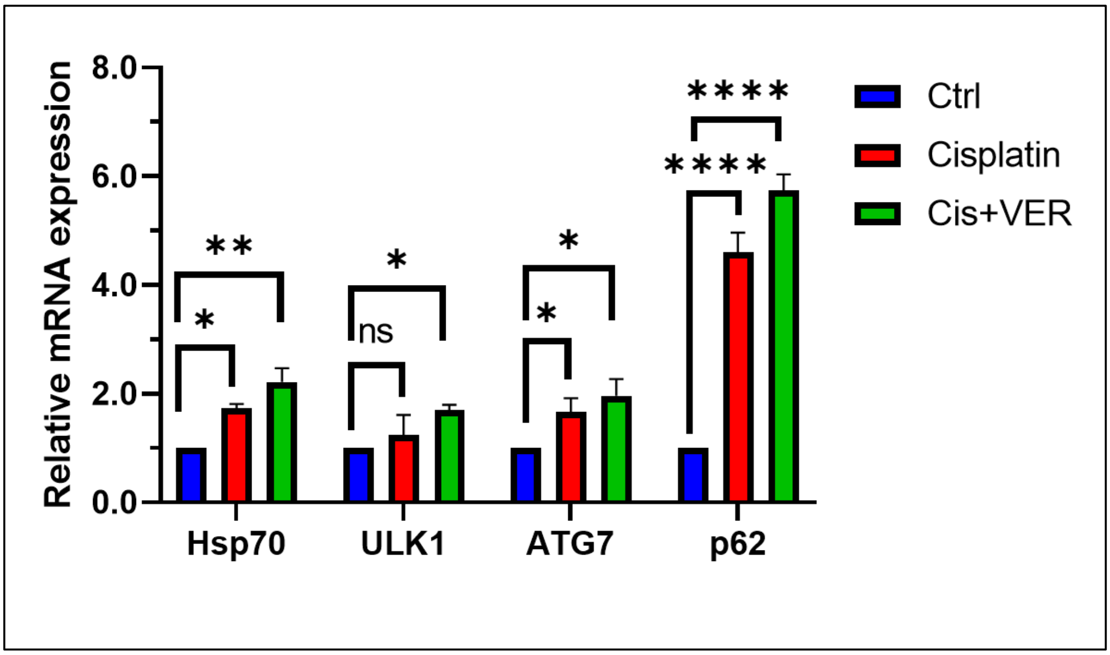

Figure S5. *Cisplatin, alone or in combination with VER-155008, promotes the transcriptional upregulation of Hsp70 and autophagy-related genes in A549 tumor cells.*

A549 tumor cells were treated with Cisplatin (10 µM) alone or in combination with VER-155008 (10 µM) for 48 hours. Afterwards, mRNA of tumor cells was extracted, converted to cDNA and subjected to RT-PCR using primers for Hsp70, ULK1, ATG7 and p62. Values are the means ± SEM from three independent experiments; * $p < 0.05$, ** $p < 0.01$, *** $p < 0.001$; **** $p < 0.0001$.

Figure S6. *Colony formation assay plates*

A549 cells plate

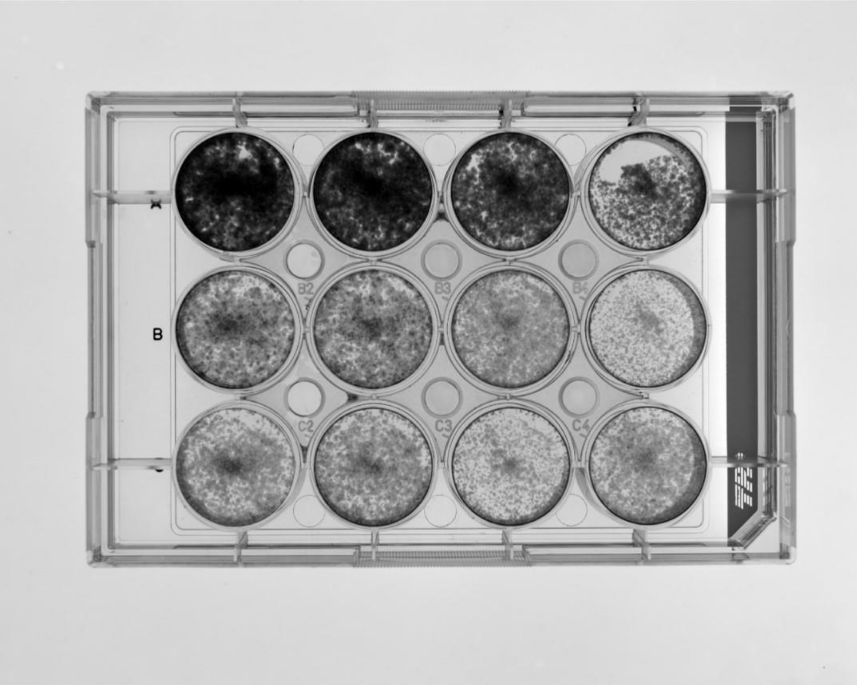

H1299 cells plate

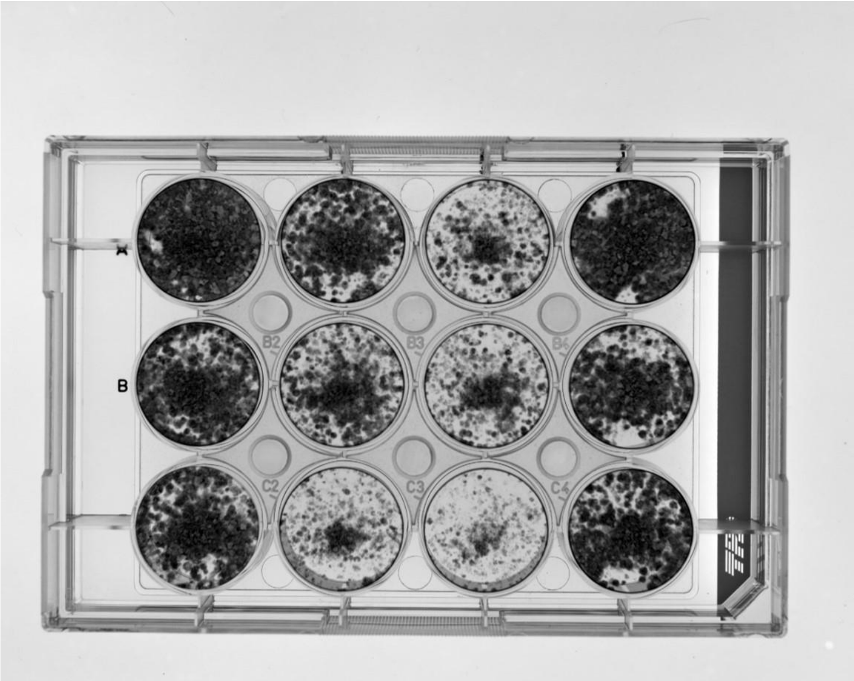

Supplement: Supplementary file 1 [file ijms-25-09090-s001.zip › ijms-3128324-supplementary.pdf]
